# Distinct Survival, Growth Lag, and rRNA Degradation Kinetics during Long-Term Starvation for Carbon or Phosphate

**DOI:** 10.1128/msphere.01006-21

**Published:** 2022-04-20

**Authors:** Yusuke Himeoka, Bertil Gummesson, Michael A. Sørensen, Sine Lo Svenningsen, Namiko Mitarai

**Affiliations:** a The Niels Bohr Institute, University of Copenhagen, Copenhagen, Denmark; b The Universal Biology Institute, University of Tokyo, Tokyo, Japan; c Department of Biology, University of Copenhagen, Copenhagen, Denmark; University of Iowa

**Keywords:** *Escherichia coli*, bacterial stress response, lag time, nutrient starvation, ribosomal RNA, stable RNA degradation, stationary phase

## Abstract

The stationary phase is the general term for the state a bacterial culture reaches when no further increase in cell mass occurs due to exhaustion of nutrients in the growth medium. Depending on the type of nutrient that is first depleted, the metabolic state of the stationary phase cells may vary greatly, and the subsistence strategies that best support cell survival may differ. As ribosomes play a central role in bacterial growth and energy expenditure, ribosome preservation is a key element of such strategies. To investigate the degree of ribosome preservation during long-term starvation, we compared the dynamics of rRNA levels of carbon-starved and phosphorus-starved Escherichia coli cultures for up to 28 days. The starved cultures’ contents of full-length 16S and 23S rRNA decreased as the starvation proceeded in both cases, and phosphorus starvation resulted in much more rapid rRNA degradation than carbon starvation. Bacterial survival and regrowth kinetics were also quantified. Upon replenishment of the nutrient in question, carbon-starved cells resumed growth faster than cells starved for phosphate for the equivalent amount of time, and for both conditions, the lag time increased with the starvation time. While these results are in accordance with the hypothesis that cells with a larger ribosome pool recover more readily upon replenishment of nutrients, we also observed that the lag time kept increasing with increasing starvation time, also when the amount of rRNA per viable cell remained constant, highlighting that lag time is not a simple function of ribosome content under long-term starvation conditions.

**IMPORTANCE** The exponential growth of bacterial populations is punctuated by long or short periods of starvation lasting from the point of nutrient exhaustion until nutrients are replenished. To understand the consequences of long-term starvation for Escherichia coli cells, we performed month-long carbon and phosphorus starvation experiments and measured three key phenotypes of the cultures, namely, the survival of the cells, the time needed for them to resume growth after nutrient replenishment, and the levels of intact rRNA preserved in the cultures. The starved cultures’ concentration of rRNA dropped with starvation time, as did cell survival, while the lag time needed for regrowth increased. While all three phenotypes were more severely affected during starvation for phosphorus than for carbon, our results demonstrate that neither survival nor lag time is correlated with ribosome content in a straightforward manner.

## INTRODUCTION

The amount of protein production machinery in a bacterial cell is tightly regulated during exponential growth, which is important for the proper allocation of the available resources to maximize protein synthesis and, thereby, growth rate (1, 2). The increased demand for protein synthesis capacity at higher growth rates can be appreciated as a linear increase in the concentration of ribosomes (estimated by the RNA/protein ratio) as a function of the steady-state exponential growth rate (3, 4). This concept was recently extended to consider relatively slow exponential growth rates obtained in chemostats by limiting a specific component of the growth medium (5). There, it was found that the RNA/protein ratio is about 2-fold lower under phosphorus-limited growth than in the carbon-limited case for a given growth rate. Since more than 60% of cellular phosphate is found in rRNA, it is not surprising that cells would have evolved mechanisms to reduce ribosomes to a minimal number under phosphorus-limited growth conditions. This finding also indicates that cells have more ribosomes than they need for protein synthesis in the carbon-limited case, which was confirmed by an accumulation of mRNA-free ribosomes under this growth condition (5). An overcapacity of ribosomes has been noted previously and was proposed to enable a rapid response to a nutrient upshift (6, 7).

Upon severe starvation where the increase of biomass comes to a halt, ribosomes are known to be subject to degradation. This has been demonstrated in various conditions, including carbon starvation (8–12) and phosphorus starvation (12–14). In a comparative short-term study, we showed that 80 min after abrupt starvation introduced by filtration of bacteria into a growth medium lacking a particular nutrient type, 10% of rRNA was degraded for carbon starvation, whereas ∼40% was degraded during the first 1 to 2 h of phosphate starvation (12). In qualitative agreement with this observation, others have reported that 12 to 24% of ribosomes were degraded after 3 to 4 h of carbon starvation (10, 11), and about 60% were degraded after 24 h (11), while for phosphorus starvation ribosome degradation was more severe, resulting in 50 to 80% degradation after 12 h of starvation (14).

Recent studies of carbon starvation report an exponential decrease in cell viability during several weeks of starvation after exhaustion of the carbon source in a minimal medium (15, 16). However, the potential effects of cellular ribosome content on the physiological behavior of the cells, such as their viability and the lag time, i.e., the time required for starved cells to commence growing after the addition of fresh medium, has not been explored under long-term starvation. In this paper, we studied the effects of long-term starvation (up to 28 days) for carbon and phosphorus on ribosome levels, survival kinetics, and growth lag time.

## RESULTS

### Quantification of rRNA in long-term-starved Escherichia coli cells.

In order to accurately determine the relative rRNA levels over the long-term starvation period examined here, it was necessary to first establish a quantification method that allowed a meaningful comparison of relative RNA levels in culture aliquots harvested on different days. Since the efficiency of RNA extraction can vary from sample to sample, it is necessary to normalize the measured levels of the RNA of interest to a suitable reference RNA. Given the month-long duration of the starvation period, no endogenous RNAs could be expected to remain at the same level throughout the experiment. A whole-cell spike-in method is useful for such circumstances (17) and is carried out by spiking each experimental sample with a small fixed relative volume of reference bacterial cells, followed by normalization of the level of the RNA of interest to one or more RNA species that are highly abundant in the spike-in cells and absent in the experimental samples. We used a spike-in culture of E. coli that overexpresses the rare tRNA tRNA^selC^, which is undetectable in the experimental samples (18, 19). Aliquots of a spike-in culture were stored in RNAlater, and for each RNA harvest day, a frozen aliquot was thawed and mixed with the sample culture, and relative rRNA levels were reported as the ratio of rRNA to tRNA^selC^.

**FIG 1:**
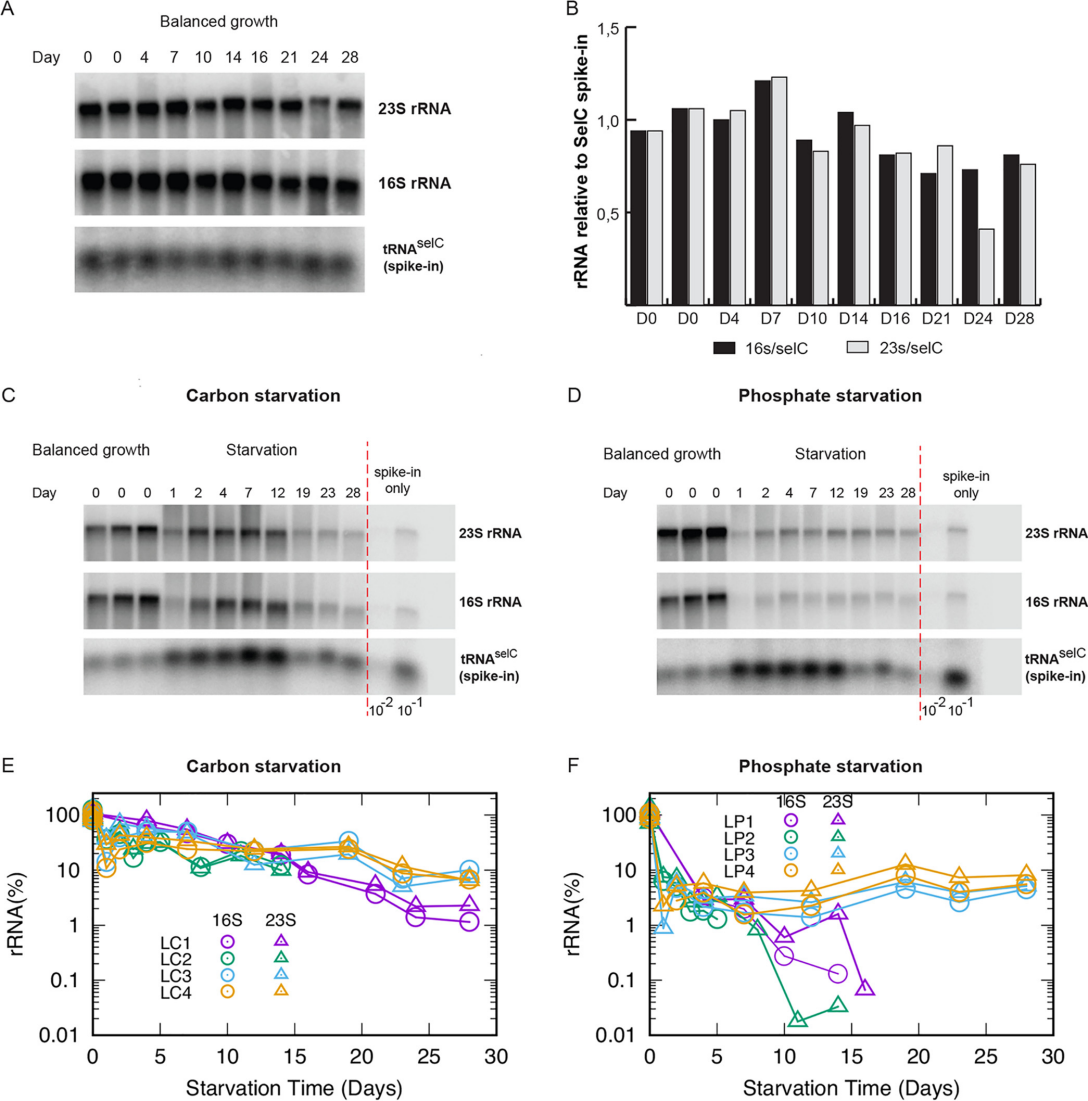
Quantification of rRNA in cultures undergoing long-term starvation. (A) Northern blot of total RNA from samples harvested during balanced growth. Day 0 indicates the start of the experiment, and days 4, 7, 10, 14, 16, 21, 24, and 28 indicate the number of days the spike-in cells were stored prior to mixing with a fresh aliquot of cells in balanced growth. The resulting blot was probed for 23S, 16S, and the spike-in-cell-specific tRNA^selC^ as indicated. (B) The levels of rRNA (16S, black bars; 23S, gray bars) were quantified by normalizing to tRNA^SelC^ from the spike-in cells and shown relative to the average of the two RNA samples harvested on day 0. (C) Northern blot of total RNA from cultures starved for carbon (days 1, 2, 4, 7, 12, 19, 23, and 28 indicate the number of starvation days, while day 0 indicates samples harvested in triplicate while the culture was still growing exponentially, before the carbon source was depleted). (D) Northern blot of total RNA from cultures starved for phosphate (days 1, 2, 4, 7, 12, 19, 23, and 28 indicate the number of starvation days, while day 0 indicates samples harvested in triplicate while the culture was still growing exponentially, before the phosphate source was depleted). For both Northern blots in panels C and D, the right panel shows 1/100 and 1/10 dilutions of samples harvested from the spike-in cells. The blots were probed for 23S rRNA, 16S rRNA, and tRNA^SelC^ as indicated. (E) The plots show 16S and 23S rRNA levels relative to balanced growth during carbon starvation; (F) the quantification of 16S and 23S rRNA during phosphate starvation. Independent biological replicates are labeled as LC1 to LC4 for carbon starvation and as LP1 to LP4 for phosphate starvation. In the later time samples for phosphorus starvation, the rRNA level in the culture sometimes became too low to distinguish from the contribution from spike-in cells. Those data points are not shown (see Materials and Methods).

To verify that the frozen stocks of spike-in cells carrying tRNA^selC^ could be used for normalization during a long-term experiment, we took advantage of the fact that ribosome content is linearly related to growth rate under steady-state exponential growth (3, 4), and thus, the rRNA per optical density (OD) unit of cells should be the same in E. coli cultures growing at the same steady-state growth rate, even if measured on separate cultures on different days. To test the method over the course of 28 days, we grew a culture of wild-type E. coli K-12 every few days to balanced growth with a doubling time of around 54 min. Culture aliquots were mixed with aliquots of the stored spike-in culture at a fixed ratio of 0.05 spike-in culture to experimental culture, based on OD units. RNA was purified from the cell mixture and stored, and at the end of the 28-day period, we ran a Northern blot analysis of all the samples to test whether the ratio of rRNA to tRNA^selC^ was constant. Figure 1A shows an example of such a Northern blot, and Fig. 1B shows that the rRNA/spike-in ratio was indeed fairly constant over the 28-day period, varying with a standard deviation of 16% for 16S rRNA/tRNA^selC^ and 23% for 23S rRNA/tRNA^selC^. The additional variation in 23S rRNA is caused by the sample from day 24, where extensive degradation of 23S rRNA in the sample is noticeable on the Northern blot. No systematic trends (for example, an increase of rRNA over time due to the degradation of tRNA^selC^ in the frozen stock) could be observed. Thus, a frozen whole-cell spike-in culture allows quantification of relative rRNA levels over at least a 28-day period.

To monitor the long-term effects of starvation for carbon or phosphorus, we grew cultures to balanced growth, after which the bacteria were subjected to starvation for the carbon source (glucose) or phosphorus source (potassium diphosphate) by transfer to MOPS (morpholinepropanesulfonic acid) low-carbon (LC) or MOPS low-phosphorus (LP) medium. The cultures remained in the shaking water bath for up to 28 days and were sampled for measurements of rRNA content, cell viability, and growth lag time as described in detail in Materials and Methods. In both low-nutrient media, the cultures reached an OD at 436 nm (OD_436_) of 1 to 2 within 8 to 10 h and then entered the stationary phase due to the lack of the limiting nutrient.

To determine the rRNA levels in the starved cultures, culture aliquots were mixed with known quantities of the frozen stock spike-in as described above for cultures in balanced growth. Examples of the resulting Northern blots are presented in Fig. 1C and D. (Full blots are shown in Fig. S2). The results of four independent month-long experiments are shown in Fig. 1E and F for the carbon and phosphate starvation, respectively.

10.1128/msphere.01006-21.2 FIG S2 Full image scan of Northern blots from Fig. 1C and D in the main article. (a and b) Blots of total RNA from cultures starved for carbon (a) and for phosphate (b) probed for 23S rRNA (left panel) and 16S rRNA (right panel). Samples harvested from the exponentially growing cultures before the carbon or phosphate source became depleted are depicted as day 0. Control precultures in balanced growth are depicted by –; these samples were not used to compute the reported rRNA decay in starvation. Days 1, 2, 4, 7, 12, 19, 23, and 28 indicate the number of starvation days. For all blots, the two last lanes show 1/100 and 1/10 dilutions of samples harvested from the spike-in cells. Download FIG S2, JPG file, 0.8 MB. Copyright © 2022 Himeoka et al. 2022 Himeoka et al. https://creativecommons.org/licenses/by/4.0/ This content is distributed under the terms of the Creative Commons Attribution 4.0 International license.

In the carbon starvation case (Fig. 1E), the levels of intact rRNA per culture volume decreased to 10 to ∼20% of the value during balanced growth after approximately 2 weeks. In the final 2 weeks, the rRNA concentration decreased monotonically to 1% in LC1, but remained around 10% in LC3 and LC4. We kept the starvation culture only up to day 14 for LC2 and LP2 for sampling the culture in a short time interval. Thus, the data are not presented after the 14th day for LC2 and LP2. In contrast, rapid and extensive rRNA degradation was observed from the onset of phosphorus starvation (Fig. 1F). Within the first day of starvation, the rRNA concentration had dropped below 10% for LP2, 3, and 4 (the sample of day1 was not harvested in LP1). After this quick drop, the decrease continued and the amounts of rRNAs dropped below the level at which we could reliably measure it on the Northern blots after 10 days for two replicates (LP1 and LP2). In contrast, the rRNA level remained roughly constant after the quick drop on the first day for the other two replicates (LP3 and LP4). One limitation of our whole-cell spike-in approach is that the rRNA from the spike-in cells, which contributes insignificantly to the total rRNA when mixed into growing cultures at a 1:20 ratio, overwhelms the rRNA contribution from the starved cultures once a large percentage of their rRNA has been degraded. Relative rRNA levels below ∼5% are therefore close to the detection limit of our method and are simply evidence that the rRNA levels are “very low” at the qualitative level. Therefore, the qualitative difference of the behaviors of rRNA level between the first (LP1 to LP2) and the second (LP3 to LP4) groups of replicates is not substantial. In conclusion, cultures that enter the stationary phase due to the lack of phosphate, an integral part of the RNA backbone, show much more extensive rRNA degradation than cultures starved for the carbon and energy source glucose, even several days or weeks into starvation.

### Distinct survival kinetics of E. coli during carbon and phosphorus starvation.

**FIG 2:**
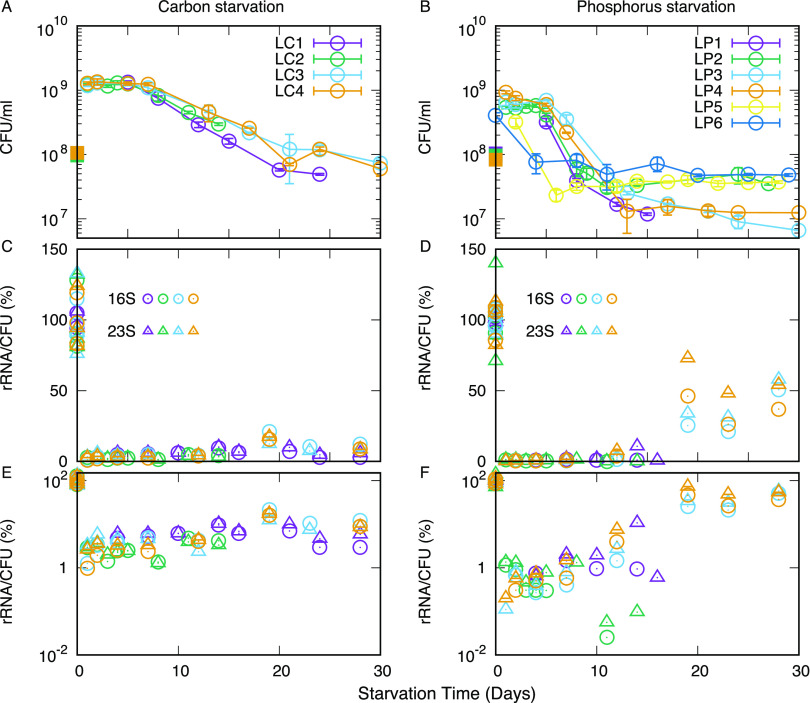
Survival kinetics of E. coli during long-term starvation and the population-averaged rRNA content. (A and B) Time course of CFU/mL for (A) the carbon starvation case and (B) the phosphorus starvation case. Independent measurements are labeled as LC1 to LC4 for carbon starvation and as LP1 to LP6 for phosphate starvation (amounts of rRNAs were not quantified for LC5 and LC6). Square symbols on day 0 show the estimated CFU/mL at the time of RNA harvest from exponentially growing cultures, calculated based on OD_436_. (C and D) The relative levels of 16S and 23S rRNA per surviving (colony-forming) bacterium. (E and F) rRNAs per colony-forming cell are plotted on a semi-log scale. The error bars in panels A and B indicate the unbiased standard error.

We wondered how the distinct rRNA degradation dynamics related to cell survival during starvation for the two different types of nutrients. The kinetics of cell death under starvation was studied by measuring the ability of cells from the starved cultures to form colonies within 2 days on rich-medium agar plates. The number of viable cells (CFU) per culture volume is depicted in Fig. 2A for the carbon-starved case and Fig. 2B for the phosphorus-starved case as a function of the time the cells had remained under starvation conditions prior to plating on rich medium. In both cases, viability remained somewhat constant for the first 5 days of starvation. After that, carbon and phosphorous starvation resulted in remarkably different survival kinetics. The concentration of viable cells in carbon-starved cultures showed a continuous decay, consistent with recent results of carbon-starvation by glycerol depletion (16, 20). On the other hand, cell viability showed a rapid ∼12-fold drop around day 5 to 6, and then the decreases slowed down or even stopped in phosphorus starvation. The behavior of the phosphorus-starved cultures is similar to that reported for E. coli outgrowth from complex LB medium, containing a stationary phase and a death phase, followed by a long-term stationary phase (21).

To illustrate the average decrease in ribosome levels per viable cell, the percentage of rRNA/CFU relative to cultures in balanced growth are shown in Fig. 2C and D. In the case of carbon starvation, the rRNA/CFU ratio fluctuates from a few percent to approximately 20% of the level in balanced growth throughout the starvation period (Fig. 2C and E). The magnitude of the initial drop is at least partly caused by the increase in CFU that occurs without a concomitant increase in biomass when cells undergo reductive division upon entry to the stationary phase (22–25). Since reductive cell divisions result in a reduction of the average cell volume, the actual drop in the average intracellular concentration of ribosomes may be 2- to 4-fold less than the relative rRNA levels depicted in Fig. 2C and E would suggest.

In the phosphate starvation case, the drop of rRNA/CFU (Fig. 2D and F) early on is significantly more rapid and severe than the carbon starvation case, showing more than a 10-fold decrease already after a day, and decreasing to a few percent of the original value by day 4. Again, the fluctuations seen far into phosphate starvation are likely to be explained by imprecise measures of the very low levels of rRNA relative to the contribution from the spike-in cells. Note that though both rRNA levels and cell viability tend to decrease over time, the rate of decrease is different, and this results in a somewhat increasing trend of rRNA/CFU after day 5 (Fig. 2). We suspect this trend might reflect that some full-length rRNA molecules from nonculturable cells in the culture are detected on the Northern blots. However, it should also be noted that the rRNA level itself is very low and close to the detection limit for later days.

The finding that phosphorus-starved cells on average survive with many fewer ribosomes per viable cell than carbon-starved cells suggests that more ribosomes than the necessary minimum are maintained in carbon-starved cells also during long-term starvation.

### Growth lag time after the stationary phase depends on the type of starvation.

The time interval from when starved cells are placed under growth-permitting conditions and until growth is actually observed, the lag time, is a measure of the physiological readiness of the cells to resume active conversion of nutrients into bacterial biomass. Several reports suggest that lag times generally increase with the duration of the starvation time (26–29), although a unifying molecular mechanism underlying this correlation has not been identified. Since protein synthesis activity by the ribosomes is a prerequisite for cell growth, one could naively expect that a progressive loss of ribosomes during starvation could be the underlying cause of the positive correlation between starvation time and the subsequent growth lag time.

**FIG 3:**
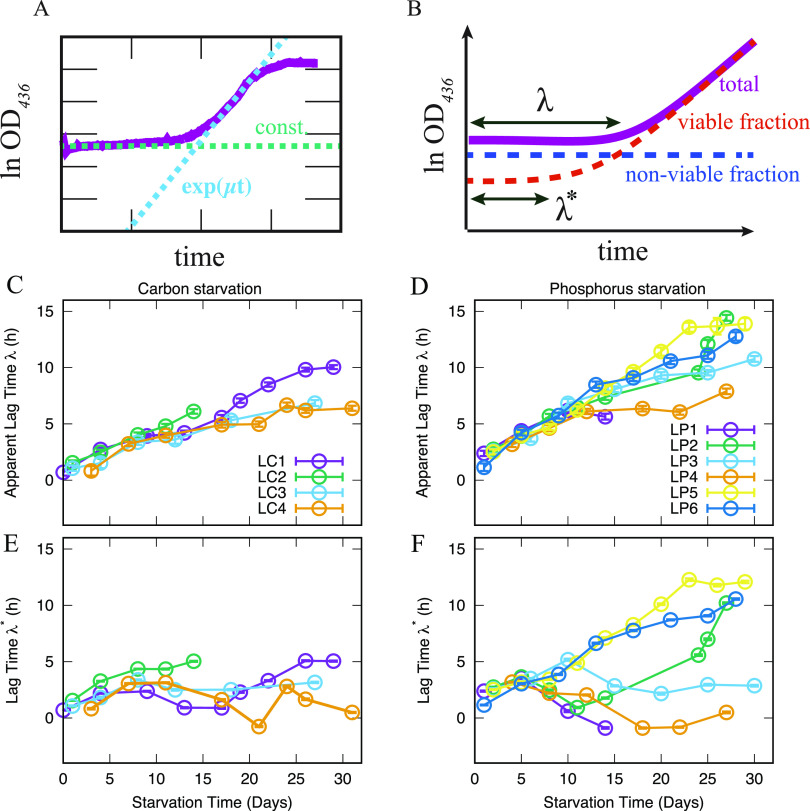
Lag time measurements after carbon or phosphorus starvation. (A) An example of the time series of resuscitation and fitting parameters. The growth curve of the bacterial culture is shown in magenta. The lag phase is fitted by a constant function *f* (dashed green line), and the exponential phase is fitted by an exponential function *g* (dashed blue line). The value of *f*, the slope of *g*, and the intersection point of *f* and *g* correspond to the initial OD_436_ value (*N_0_*), the specific growth rate (*μ*), and the lag time (*λ*), respectively. (B). An illustration of how the effect of nonviable cells is subtracted from the average lag time. We decomposed the total population into viable (red) and nonviable (blue) fractions based on the measured cell viability (CFU/mL) and recalculated the lag time for the viable fraction (λ*). (C and D) The average apparent lag time *λ* for (C) carbon starvation and (D) phosphorus starvation. (E and F) The lag time λ* after subtraction of the effect of nonviable cells for (E) carbon starvation and (F) phosphorus starvation. The error bars are the unbiased standard error of the mean (The number of samples taken to measure OD curves used to estimate the lag time for one biological replicate is typically more than 80 per time point. For the propagated errors, see Materials and Methods). For panel C to F, each biological replicate is shown separately.

We measured the lag time of populations of E. coli as a function of starvation time in medium lacking carbon or phosphorus. At each measurement point, an aliquot from the starved culture was diluted into fresh MOPS minimal medium supplemented with 0.2% glucose or 1.32 mM potassium phosphate, respectively, and distributed in 88 wells of a microtiter plate, and the temporal evolution of optical density (OD_436_) was monitored using a microtiter plate reader. The obtained growth curves were fitted by a constant function and an exponential function, as shown in Fig. 3A. From the fitting, the maximum growth rate *μ* and the lag time (the time at which the constant function and the exponential function intersects in Fig. 3A) were computed for each growth curve. The average specific growth rate and lag time were then computed from the growth curves obtained for each measurement point. Example time courses are shown in Fig. S3. We call the lag time obtained in this way the apparent lag time (*λ*). The average growth rate obtained from the fitting is plotted in Fig. S4. Figure 3C and D show the apparent lag time *λ* as a function of the starvation time for carbon starvation and phosphorus starvation, respectively. Consistent with the literature cited above, the duration of the apparent lag time *λ* increased monotonically with the starvation time for both types of starvation. Furthermore, phosphate starvation had a more pronounced effect on the growth lag than carbon starvation, as cultures starved for phosphate showed a growth lag of several hours already after 1 day of starvation, while a similar lag time was first observed after ∼4 days of carbon starvation. We also observed a stronger dependence of growth lag on starvation time for the phosphate-starved culture throughout the 30 days of starvation, as the slope of the function was steeper for phosphate starvation (0.41 h/day) than for carbon starvation (0.33 h/day).

10.1128/msphere.01006-21.3 FIG S3 All time courses of OD_436_ used to estimate the lag time. The purple lines are the actual data. The green and the blue lines represent the best fits for the lag phase and the exponential phase, respectively. The data are from the measurement of the second replicate of a phosphorus-starved culture (LP2 in the main text) at the 27-day starvation point. Download FIG S3, EPS file, 2.8 MB. Copyright © 2022 Himeoka et al. 2022 Himeoka et al. https://creativecommons.org/licenses/by/4.0/ This content is distributed under the terms of the Creative Commons Attribution 4.0 International license.

10.1128/msphere.01006-21.4 FIG S4 (A and B) The average doubling time during the regrowth from the cultures of (A) carbon starvation and (B) phosphorus starvation. Error bars indicate the standard error. Download FIG S4, EPS file, 0.07 MB. Copyright © 2022 Himeoka et al. 2022 Himeoka et al. https://creativecommons.org/licenses/by/4.0/ This content is distributed under the terms of the Creative Commons Attribution 4.0 International license.

It should be noted that the measurement of apparent lag time *λ* is affected by the drop in the concentration of viable cells that occurred during starvation because it takes more time for the OD_436_ to increase during regrowth if only a small fraction of the cells that contribute to the initial OD_436_ value can resume growth. We tried to consider this effect under the simplest assumption that all the viable cells resume growth after the same lag time, which will be variable at the single-cell level in reality (see Fig. 3B). Following this view, we decomposed the OD_436_ value into two parts, namely, the viable fraction and the nonviable fraction based on the value of CFU/mL in the starved culture, the volume of sample transferred to the microtiter plate, and the OD_436_ curves after dilution into fresh growth medium. We further assumed that the OD_436_ of viable cells increased at growth rate *μ* after a certain growth lag from the inoculation time, while the nonviable fraction never resuscitated. Under this assumption, we computed the lag time λ* where the death-effect was subtracted. A schematic description of this calculation is shown in Fig. 3B.

The calculated lag time λ* is shown in Fig. 3E and F for carbon and phosphorus starvation, respectively. While the overall correlation between the starvation time and λ* persists, the subtraction of the nonviable cells makes the data much noisier. In particular, the steep drop in cell viability on day 5 to ∼10 in LP1, LP2, and LP4 leads to a decrease of the corrected lag time. In addition, the correction even results in a negative lag time for LP1 and LP4. We attribute this problem to the correction method. First, the subtraction method disregards the population heterogeneity of the lag time of individual cells and groups them into simply “viable” and “nonviable.” This simplification leads to the underestimate of the lag time λ* (see Text S1 section 2 for more detail). Second, the number of viable cells and the individual cell’s lag time may be correlated, since any possible cell lysis can change the available nutrients and other chemical conditions in the culture (cf. 16).

## DISCUSSION

We have carried out long-term starvation experiments with carbon-limited and phosphate-limited E. coli cultures to study the long-term change in the rRNA level, viability, and lag time of bacteria starved for different nutrient types.

Under both starvation conditions, the concentration of full-length rRNA in the culture decreased with time. The rate of decrease under phosphorus starvation was appreciably higher than that under carbon starvation. This observation is consistent with data from recent short-term starvation experiments (12, 16), thereby extending the validity of those observations to include rRNA degradation in the late-stationary and death phases. Given that RNA is a phosphorus-rich polymer and that most RNA is found in the ribosomes, the more rapid rRNA degradation under phosphorus starvation might be interpreted as cells using the ribosomes as a reservoir of phosphorus. Phosphate starvation leads to a rapid and severe depletion of nucleotide triphosphates, including the cellular energy currency ATP (30). Nucleotides released from rRNA could therefore support essential cellular functions, in particular protein synthesis, enhancing stress survival in the phosphorus-starved cells (31). On the other hand, the nucleotides stored in rRNA may not benefit carbon-starved cells to the same degree, as the reduction in ATP levels is slow and gradual during carbon starvation (32, 33). The molecular pathway for rRNA degradation has been discerned (11), but intriguingly, the known regulatory mechanisms do not predict the observed differences in the rates of ribosome degradation under starvation for carbon and phosphorus. While for carbon starvation we observed that the decrease lasted for 28 days, due to the rapid degradation dynamics, we could only quantify the rRNA concentration under phosphorus starvation for up to 16 days in two cases. Taking the observation that a number of mutations accumulate during long-term starvation in LB medium (34) into account, it is likely that mutations accumulate also in our starvation experiments, and it would be interesting to investigate whether the type of nutrient depletion that initiated the starvation impacts the set of genes that accumulate mutations in the starved cultures. The distinct behavior of rRNA levels in our replicate cultures, especially in the later stages of phosphorus starvation, are likely due to the difficulty of precisely quantifying low rRNA levels by our method. We refrained from using more sensitive RNA detection methods, such as reverse transcription-quantitative PCR (qRT-PCR), for detection of rRNA late in starvation, because qRT-PCR would not allow us to distinguish full-length rRNA molecules from cleaved degradation products. While the main RNA species detected in the Northern blots were the full-length species, the resolution of fragments was not very high with our agarose gels to quantify specific rRNA fragments (Fig. S2). We therefore refer readers to, e.g., references 35 and 36 for more information on specific rRNA fragments formed in carbon-starved cells. Finally, we also consider the possibility that the different behaviors of the replicate cultures could be due to accumulation of different mutants in the long-term-starved cultures.

The survival kinetics showed distinct behaviors depending on the limiting nutrient. Under carbon starvation, viability decreased over the starvation period, while cell viability remained approximately constant after an early steep drop (over 5 to 15 days) under phosphorus starvation. Recently, the maintenance energy requirement (37) was shown to set the death rate of cells under carbon starvation (20). Given the similar death kinetics, cell death under carbon starvation is also likely led by the same mechanism in the present experiments. In contrast, the pattern of viability loss under phosphorus starvation is not predicted by the maintenance energy requirement theory. A possible attractive hypothesis to explain the extensive loss of viability a few days into phosphorus starvation is that a certain threshold level of ribosomes per cell is necessary for viability and that the average bacterium crossed this threshold during the first starvation days.

Far into the starvation period, culturable bacteria under phosphorus starvation showed much lower rRNA levels (rRNA/CFU) than the carbon starvation case. Thus, the carbon-starved cells appear to keep substantially more ribosomes per cell than what is the necessary level for viability, while the ribosomes per cell in the phosphorus-starved cells may be close to the critical level. The storage of inactive ribosomes under the carbon-limited condition has been suggested to aid E. coli in rapidly accelerating the growth rate during nutrient upshift (5, 6).

We also measured the regrowth lag time to study if the decreasing rRNA level during the starvation period is reflected in the time it takes bacteria to resume growth upon replenishment of nutrients. The average apparent lag time *λ* showed a linearly increasing trend with the starvation time. The delay in regrowth, as cultures have starved for longer, could result from an increasing metabolic or regulatory delay in the individual viable cells, a decreasing viable fraction of the biomass that contributes to the cultures’ optical density, or both. The increasing trend of lag time with starvation time may suggest the existence of a metabolic or regulatory delay in individual viable cells that increases during long-term starvation for carbon or phosphorus. Furthermore, the difference in the steepness of the increase in growth lag with increased starvation time implies that differences in the type of nutrient deficiency that induced starvation are persistently reflected in the state of the cells for at least a month, as the amount of rRNA, the duration of the lag time, and the fraction of viable cells stayed qualitatively and quantitatively different between the two starvation conditions. However, we did not observe a clear correlation between the lag time and levels of rRNA per cell for either starvation condition.

We wish to emphasize that all our measurements were carried out at the population level, not at the single-cell level. The calculated value of rRNA per viable cell was relatively constant after the initial drop, but that does not necessarily mean that the amount of rRNA in individual cells remained constant. As an extreme scenario, the amount of rRNA may decrease over time for some cells, while other cells may even synthesize new rRNA during starvation. Also, for the lag time measurement, there were roughly 10^6^ to 10^7^ cells in each well of the microtiter plates when the fresh medium was added. As shown by Levin-Reisman et al. (29) and Moreno-Gámez et al. (38), the duration of the lag time differs among individuals. If a small fraction of the cells had a relatively short lag time, their offspring would dominate the whole subculture. In other words, our measurements are likely to reflect how the shortest end of the lag time distribution changes with starvation time.

Phenomenological modeling of bacteria under starved or substrate-limited conditions has been attracting attention recently (39, 40). The model proposed by one of the authors of the present paper (40) predicts that the lag time increases in a square-root manner with the starvation time. The model prediction fits some experimental data (28, 29), but the correspondence is not clear for the data shown in the present paper. There are several scenarios to be tested. First, the population-level measurements could mask important aspects of the lag time behavior at the single-cell level. Second, the functional form of the lag time could depend on the specific setup of the starvation experiment. All of the above-cited starvation experiments were carried out in LB medium, and at low temperatures for one experiment (28). These differences could affect the physiological state of the cells, leading to distinct results. Further investigation from both the experimental and theoretical sides are needed to fill the gap among those observations and the present result, which could potentially unveil universal features of the bacterial lag phase and stress responses.

As far as we know, this study is unique in that we measured the rRNA level and the lag time over a month-long starvation period under well-controlled conditions. Our results demonstrate that even at the endpoint, when the cultures had been starved for 1 month, carbon-starved cells on average kept a higher level of rRNA per cell than phosphorus-starved cells did. Similarly, after a month of starvation, the carbon-starved cells showed a shorter lag time than the phosphorus-starved cells, which may support the idea that storage of a ribosome surplus allows carbon-starved cells to respond faster to the reintroduction of nutrients to the medium (5). All in all, our results suggest that neither cell viability nor lag time after long-term starvation is a simple function of cellular ribosome content. A more stringent examination of this question would require simultaneous measurements of the ribosome level and the lag time at the single-cell level. Such experiments are therefore strongly desired.

## MATERIALS AND METHODS

### Bacterial strain and media.

The wild-type strain Escherichia coli K-12 MAS1081 (MG1655 *rph*^+^ *gatC*^+^ *glpR*^+^) was used in all growth experiments. Cultures were kept at 37°C with shaking at 190 to 220 rpm in a water bath, in morpholinepropanesulfonic acid (MOPS) minimal medium supplemented with 0.2% glucose (41). Cell growth was monitored spectrophotometrically by optical density at 436 nm (OD_436_), and cultures were grown exponentially for at least 15 generations before being exposed to starvation.

Carbon or phosphorus starvation was introduced gradually by transferring cells from balanced growth cultures to preheated MOPS medium supplemented with 0.07% glucose (designated here as MOPS low-carbon [LC] medium) or MOPS medium with 0.2% glucose but with reduced dipotassium phosphate (0.066 mM instead of 1.32 mM) (designated here as MOPS low-phosphate [LP] medium), and letting the cultures enter the stationary phase due to the lack of a carbon source (LC cultures) or phosphorus source (LP cultures).

For RNA measurements by Northern blotting, whole-cell spike-in cultures of the E. coli strain MAS1074 were used for normalization as in reference 17. MAS1074 expresses the *selC* gene encoding the rare tRNA^selC^ under the control of an isopropyl β-d-1-thiogalactopyranoside (IPTG)-inducible promoter (18). The spike-in strain was grown in MOPS medium with 0.2% glucose and 100 μg/mL ampicillin at 37°C. tRNA^selC^ expression was induced at an OD_436_ of 0.10 by adding IPTG to a final concentration of 1 mM. After 4 h of inducing conditions, the spike-in culture was aliquoted in a 1:2 ratio with RNAlater solution (Invitrogen) on ice and stored at −80°C.

### Viability measurement.

Cell viability was determined by counting the number of CFU from an aliquot of the starved cultures on Luria-Bertani (LB) agar plates. After plating, the plates were incubated at 37°C. The number of colonies was counted after one overnight incubation as well as after 2 days of incubation. The CFU did not change after 2 days of incubation as assessed by counting the plates again after 3 days of incubation and obtaining identical numbers. Serial dilutions were performed so that the number of colonies was 100 to ∼200 per plate, while it occasionally dropped below 50, especially in the phosphorus-starvation cultures. The average values and standard deviations reported for viability measurements were based on at least four plates.

### Spike-in normalization and RNA extraction.

For rRNA measurements, bacterial culture samples were harvested by mixing an aliquot of the culture with a 1/5 volume of a stop solution composed of 5% water-saturated phenol in ethanol at 0°C (42). Prior to total RNA extraction, a volume of spike-in culture corresponding to 5% of the experimental sample based on OD was added. Total RNA was extracted from the spiked sample by hot phenol as described in reference 17.

### Northern blots and rRNA quantification.

Northern blots were performed as described in (17). Briefly, 5′-end-labeled oligo-DNA probes (γ-[32P]-ATP; PerkinElmer) complementary to a sequence in the 16S rRNA, 23S rRNA, or SelC tRNA were used to detect the immobilized RNA on the membranes. Probe sequences are listed in Table S1. The radioactivity present in specific bands was measured on a Typhoon phosphorimager FLA7000 (GE Healthcare) at a 100-μ resolution and quantified using ImageQuant TL 8.2. The quantified intensity of each rRNA band was first adjusted to account for the contribution from the spike-in cells using a lane on the same Northern blot that was loaded with spike-in cells only. More precisely, in addition to a high concentration of the overexpressed tRNA^selC^, the spike-in cells also contribute small amounts of all other cellular RNAs to the samples. Since rRNA contributions from the spike-in cells are proportional to the tRNA^SelC^ contribution, we subtracted the corresponding value from the measured rRNA levels. The corrected rRNA signal intensities were then divided with the tRNA^selC^ signal from the same lane to give the normalized rRNA level. For Fig. 1B, this ratio is plotted relative to the average of the two samples harvested on day 0, while for Fig. 1E and F, the ratio is plotted relative to the average of measurements from the culture prior to starvation. While changes in OD provide a clear measure of the growth of bacterial cultures in the steady state, where all cell constituents grow with the same rate, changes in OD are complex to interpret upon disruption of the steady state, e.g., by starvation (12, 43). Accordingly, the correlation between OD measurements and bacterial concentration (CFU/mL) was good for exponentially growing cultures but poor for the starved cultures (Fig. S1).

10.1128/msphere.01006-21.1 FIG S1 (A and B) The ratios of OD_436_ to the concentration of viable cells (CFU/mL) for (A) carbon starvation and (B) phosphorus starvation. Each dot was obtained by taking the ratio between OD and CFU/mL for a given data point of CFU/mL. If the OD was not measured at the same time as the CFU, the OD value was calculated by linearly interpolating the OD data at the two closest points on both sides of the CFU data point. Download FIG S1, EPS file, 0.07 MB. Copyright © 2022 Himeoka et al. 2022 Himeoka et al. https://creativecommons.org/licenses/by/4.0/ This content is distributed under the terms of the Creative Commons Attribution 4.0 International license.

10.1128/msphere.01006-21.5 TABLE S1 The probe sequences used for Northern blots. Download Table S1, DOCX file, 0.01 MB. Copyright © 2022 Himeoka et al. 2022 Himeoka et al. https://creativecommons.org/licenses/by/4.0/ This content is distributed under the terms of the Creative Commons Attribution 4.0 International license.

Therefore, for experiments LC3, LC4, LP3, and LP4, the spike-in culture was added as 5% of the sample according to OD only for the steady-state samples (day 0) and the first starvation sample (day 1). The volume ratio of spike-in culture to sample culture used on day 1 was then used as a constant ratio for samples collected from day 2 to day 28, thereby disregarding changes to the OD that occurred in the late stationary phase.

For experiments LC1, LC2, LP1, and LP2, the spike-in culture volumes were added according to the samples’ OD values throughout the experiment. To allow a direct comparison between all the experiments, the spike-in signals from tRNA^selC^ on day 2 to 28 of these experiments were retrospectively corrected with a factor corresponding to the ratio between the spike-in volume added on day 1 of the starvation to the spike-in volume added according to the OD on the sampling day.

### Lag time measurements.

Each measurement day, an aliquot of the starved culture was diluted into complete MOPS minimal medium. Then, 200 μL of the diluted culture was placed in each well of a microtiter plate (TPP 96-well flat-bottom plate, or Greiner 96-well flat-bottom plate), and the optical density (OD_436_) was measured in a microtiter plate reader (FLUOstar Omega; BMG Labtech) with shaking at 37°C, every 5 min for about 24 h. The dilutions were made so that the OD_436_ of the diluted culture was above the detection limit of the plate reader, which we determined as ∼0.05. The cultures were typically diluted by 10- to ∼20-fold. Four specific wells of the microtiter plates formed droplets on the lid repeatedly and gave inconsistent OD readings. The corresponding four OD curves were therefore removed from the data sets, and average growth curves from the remaining 84 wells were computed.

### Fitting of the growth curves.

The obtained time courses of the optical density were fitted by a combination of constant line *f* and a simple exponential curve *g* to determine the growth rate in the exponential phase and the duration of the lag time in each well. The fitting regions for the lag phase (by a constant line) and exponential phase (by an exponential curve) were determined manually. The fittings were discarded if the average square difference between the fitting line and data,

$$\text{D}\;={\;\text{N}}^{-1}\sum^{\text{N}-1}{}_{i=0}(\text{log}(\text{OD}\left(t_{i}\right)\;–\;\text{max}{\left(f\left(t_{i}\right),g\left(t_{i}\right)\right)}^{2}$$

exceeded 0.1, where *t_i_* is the time after at the *i*th measurement of the OD curve, and *t*_N–1_ is the upper bound of the fitting region for the exponential phase. The lag time of each well is estimated by the fitting as the cross-point of *f* and *g*. The apparent lag time for a sample is given as the average of the lag times of all the wells. The standard error is computed by propagating the unbiased standard deviations of *f* and *g*. Calculations for subtracting the death effect from the lag time are explained in Text S1 section 1.

10.1128/msphere.01006-21.6 TEXT S1 Calculations for subtracting the death effect from the lag time (section 1) and consideration of the effect of the distributed lag time (section 2). Download Text S1, PDF file, 0.1 MB. Copyright © 2022 Himeoka et al. 2022 Himeoka et al. https://creativecommons.org/licenses/by/4.0/ This content is distributed under the terms of the Creative Commons Attribution 4.0 International license.

10.1128/msphere.01006-21.7 DATA SET S1 Original data set of rRNA quantifications. Download Data Set S1, XLSX file, 0.05 MB. Copyright © 2022 Himeoka et al. 2022 Himeoka et al. https://creativecommons.org/licenses/by/4.0/ This content is distributed under the terms of the Creative Commons Attribution 4.0 International license.
