# Supplementary figures and images for: Distinct Survival, Growth Lag, and rRNA Degradation Kinetics during Long-Term Starvation for Carbon or Phosphate
Source: mSphere. 2022 Apr 20;7(3):e01006-21. doi: 10.1128/msphere.01006-21 (PMC9241543; doi:10.1128/msphere.01006-21)

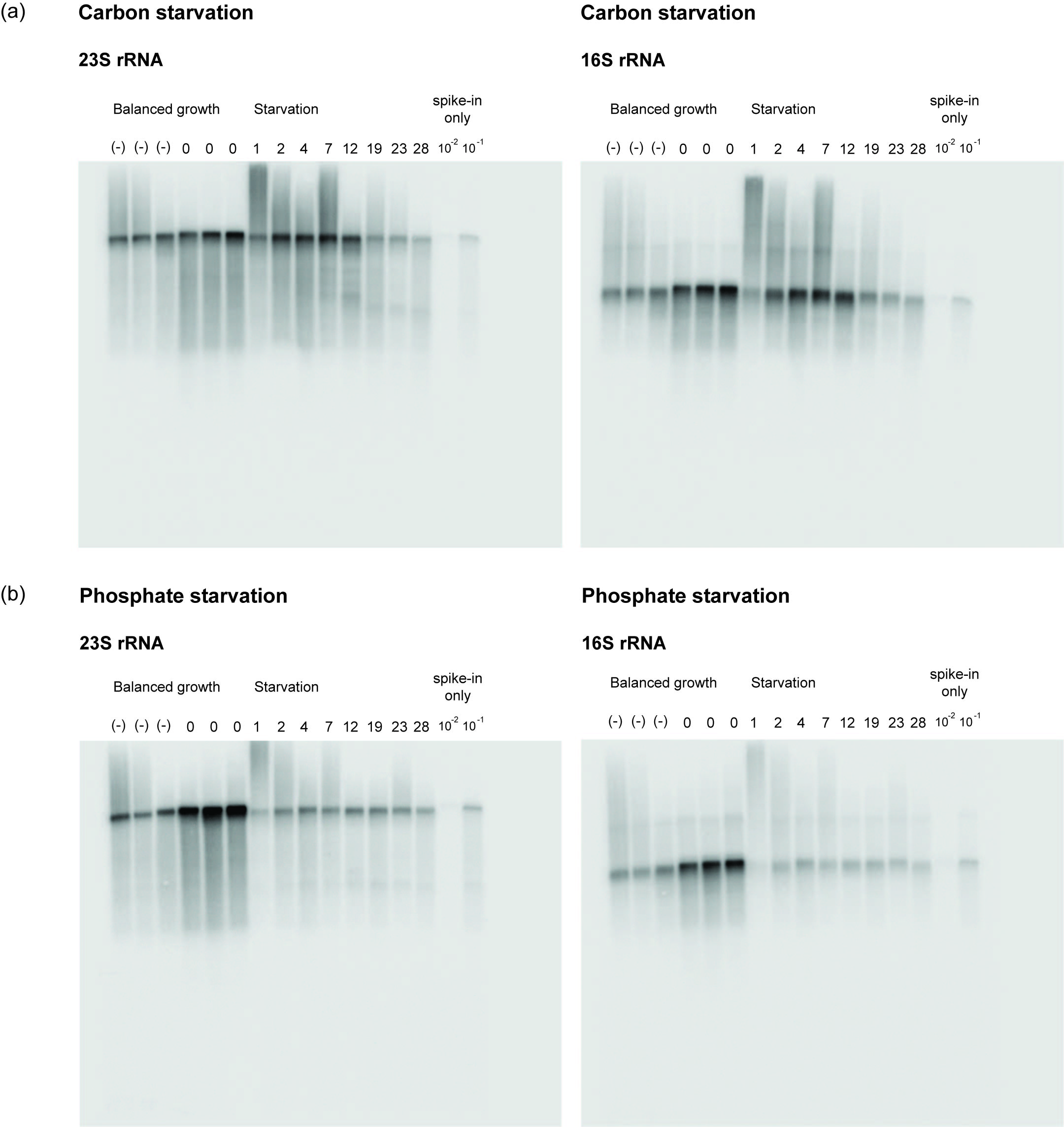

Supplement: FIG S2 [file msphere.01006-21-sf002.jpg]
