# Supplementary material for: Distinct Survival, Growth Lag, and rRNA Degradation Kinetics during Long-Term Starvation for Carbon or Phosphate
Source: mSphere. 2022 Apr 20;7(3):e01006-21. doi: 10.1128/msphere.01006-21 (PMC9241543; doi:10.1128/msphere.01006-21)
Supplement: TABLE S1 [file msphere.01006-21-st001.docx]

|  | Probe sequence |
| --- | --- |
| SelC tRNA | 5'-ATTTGAAGTCCAGCCGCC |
| 16S rRNA | 5'-AAGGAGGTGATCCAACCGCA |
| 23S rRNA | 5'-GACCCATTATACAAATACGC |

Table S1: The probe sequences used for northern blots
