# Supplementary material for: Distinct Survival, Growth Lag, and rRNA Degradation Kinetics during Long-Term Starvation for Carbon or Phosphate
Source: mSphere. 2022 Apr 20;7(3):e01006-21. doi: 10.1128/msphere.01006-21 (PMC9241543; doi:10.1128/msphere.01006-21)
Supplement: TEXT S1 [file msphere.01006-21-s0001.pdf]

Supplementary Text of "Distinct survival, growth lag, and ribosomal RNA degradation kinetics during long-term starvation for carbon or phosphate"

Yusuke Himeoka, Bertil Gummesson,
Michael A. Sørensen, Sine Lo Svenningsen, Namiko Mitarai

1 Subtraction of the death-effect from the lag time

The effects of the cell death were subtracted from the measured lag time by assuming that there are two types of the cells, namely, the viable cells and the dead cells. The viable cells start to grow at the rate μ after the time τ has passed, while the dead cells never grow. Also, we assume that there is a conversion factor from the total (viable plus dead cells) number of cells to OD and is unchanged over the starvation period. By denoting the conversion factor by a the temporal evolution of the OD curve of the resurrection after X days starvation is given as

$$
\begin{aligned}
OD_X(t) &= a(V_X + D_X), (t < \tau), &(1)\\
OD_X(t) &= a(V_X \exp[\mu(t-\tau)] + D_X), (t > \tau), &(2)
\end{aligned}
$$

where $t = 0$ is defined as the time that the cells are transferred to the fresh media and we abbreviated the wavelength index. V_X and D_X represent the number of viable cells and dead cells at time zero, respectively. Note that μ and τ can differ among measurement days and wells of microtiter plates.

Since at the first day of the resurrection measurement, R_0, the dead fraction is negligible, i.e., $D_{R_0} = 0$, we obtain the equations

$$
\begin{aligned}
OD_X(0)/OD_{R_0}(0) &= V_X/V_{R_0} + D_X/V_{R_0}, &(3)\\
OD_X(t)/OD_{R_0}(0) &= V_X \exp[\mu(t-\tau)]/V_{R_0} + D_X/V_{R_0}, (t > \tau), &(4)
\end{aligned}
$$

leading to

$$
\frac{OD_X(t)}{OD_{R_0}(0)} - \frac{OD_X(0)}{OD_{R_0}(0)} = \frac{V_X}{V_{R_0}}(\exp[\mu(t-\tau)] - 1), (t > \tau),
$$

By solving this equation about τ, we obtain

$$\tau = t + \mu^{-1} \ln\Big(V_X/V_{R_0}\Big) - \mu^{-1} \ln\left(\frac{OD_X(t)}{OD_{R_0}(0)} - \frac{OD_X(0)}{OD_{R_0}(0)} + \frac{V_X}{V_{R_0}}\right), (t > \tau), \quad (5)$$

By using CFU/ml at day X multiplied by the volume of samples transferred to the microtiter plates as V_X, we can compute τ. By definition, τ is smaller than the apparent lag time λ, and thus, the equation above holds in the exponential phase. We computed this value for each well and at each time point of the exponential phase to obtain $\tau_i(t_j)$ where i is the index of the well and j is the index of the time points. By taking the average over time and well, we obtain the subtracted lag time λ^*.

Then, the subtracted lag time λ^* at the Xth day is given as

$$\lambda^* = N^{-1} \sum_i M_i^{-1} \sum_j t_j + \mu_i^{-1} \ln\Big(V_X^{(i)}/V_{R_0}^{(i)}\Big) - \mu_i^{-1} \ln\left(\frac{OD_X^{(i)}(t_j)}{OD_{R_0}^{(i)}(0)} - \frac{OD_X^{(i)}(0)}{OD_{R_0}^{(i)}(0)} + \frac{V_X^{(i)}}{V_{R_0}^{(i)}}\right), \quad (6)$$

where N is the number of wells used for the computation of the lag time and M_i is the number of used time points of the ith well. The sum about j runs over the data points which are judged as in the exponential phase.

The standard error is computed by propagating the unbiased standard deviations of the growth rate, initial OD value, the number of transferred cells (CFU counts) with an assumption that each $\tau_i(t_j)$ follows the independent and identical distribution.

2 A subtraction method with a consideration of the lag time distribution

In the main manuscript, we have subtracted the death effect from the apparent lag time by dividing the optical density (OD) into two parts, namely, viable and non-viable fraction where all the cells in the viable fraction are considered to have the same lag time. In this section, we briefly describe this two-group subtraction method underestimates the lag time from the apparent lag time.

Let us suppose that the lag time follows the distribution function $P(\lambda)$ and the growth curve of given growth rate μ and λ is expressed as $h(t;\mu,\lambda)$ with the normalization $h(0;\mu,\lambda) = 1$. By setting the viable fraction r, the total population at time t ($t = 0$ represents the time that the starved cells are transferred to the fresh medium) is given as

$$N_0\Big((1-r) + r\int_0^\infty P(x)h(t,\mu,x)dx\Big). \quad (7)$$

Since the growth curve (Eq.(**??**)) is observed in the plate reader experiment,

$$N_0\Big((1-r) + r\int_0^\infty P(x)h(t,\mu,x)dx\Big) = h(t,\mu,\lambda) \quad (8)$$

holds with the apparent lag time λ where we assumed that N_0 and μ are correctly obtained from the experiment without subtraction because N_0 and μ are directly obtained from the OD value at $t = 0$ and the slope of the population increase in the logarithmic scale in large t region, respectively.

For instance, if we suppose the lag time distribution is the normal distribution with the peak λ^* and the variance σ^2, the subtraction method (Eq.(**??**)) leads to

$$\lambda^* = \lambda + \ln(r)/\mu + \sigma^2\mu/2.$$

Note that the lag time shown in Figure.3 (e) and (f) in the main text corresponds the case of $\sigma = 0$. We attribute the negative lag time in Figure.3 (f) to this underestimation.
